# Genetic Associations of Plasminogen Activator Inhibitor-1-Related miRNA Variants with Coronary Artery Disease

**DOI:** 10.3390/ijms252111528

**Published:** 2024-10-27

**Authors:** Yong Hyun Ha, Jung Hoon Sung, Chang Soo Ryu, Eun Ju Ko, Hyeon Woo Park, Han Sung Park, Ok Joon Kim, In Jai Kim, Nam Keun Kim

**Affiliations:** 1 Department of Biomedical Science, College of Life Science, CHA University, Seongnam 13488, Republic of Korea; hayo119@naver.com (Y.H.H.); regis2040@nate.com (C.S.R.); ejko05@naver.com (E.J.K.); aabb1114@naver.com (H.W.P.); hahnsung@naver.com (H.S.P.); 2 CHA Bundang Medical Center, Department of Cardiology, CHA University, Seongnam 13496, Republic of Korea; atropin5@cha.ac.kr; 3 CHA Bundang Medical Center, Department of Neurology, CHA University, Seongnam 13496, Republic of Korea; okjun77@cha.ac.kr

**Keywords:** coronary artery disease, plasminogen activator inhibitor-1, microRNA

## Abstract

Coronary artery disease (CAD) is one of the most common types of cardiovascular disease and can lead to a heart attack as plaque gradually builds up inside the coronary arteries, blocking blood flow. Previous studies have shown that polymorphisms in the *PAI-1* gene are associated with CAD; however, studies of the *PAI-1* 3′-untranslated region, containing a miRNA binding site, and the miRNAs that interact with it, are insufficient. To investigate the association between miRNA polymorphisms and CAD in the Korean population based on post-transcriptional regulation, we genotyped five polymorphisms in four miRNAs targeting the 3′-untranslated region of *PAI-1* using real-time PCR and TaqMan assays. We found that the mutant genotype of *miR-30c* rs928508 A > G was strongly associated with increased CAD susceptibility. In a genotype combination analysis, the combination of the homozygous mutant genotype (GG) of *miR-30c* rs928508 with the wild-type genotype (GG) of *miR-143* rs41291957 resulted in increased risk for CAD. Also, in an allele combination analysis, the combination of the mutant allele (G) of *miR-30c* rs928508 and the wild-type allele (G) of *miR-143* rs41291957 resulted in increased risk for CAD. Furthermore, metabolic syndrome and diabetes mellitus showed synergistic effects on CAD risk when combined with *miR-30c* rs928508. These results can be applied to identify CAD prognostic biomarkers among miRNA polymorphisms and various clinical factors.

## 1. Introduction

Coronary artery disease (CAD) is a form of cardiovascular disease caused by plaque accumulation on the walls of coronary arteries that supply blood to the heart. In atherosclerotic disease, these plaques eventually block blood flow to the heart. Because of this, CAD can lead to heart failure, a condition in which the heart’s ability to pump blood is reduced [1]. Risk factors related to CAD include hypertension, smoking, hyperlipidemia, and diabetes [2,3,4,5]. Several studies have reported that polymorphisms in genes related to fibrin degradation and fibrin coagulation are also associated with CAD [6,7]. In particular, some variants in the promoter region of plasminogen activator inhibitor-1 (also known as SERPINE1 or *PAI-1*), which regulates fibrinolysis, are thought to be associated with CAD sensitivity [8,9].

Micro (mi)RNAs are a class of small (18–22 nucleotides) non-coding RNAs that play important regulatory roles in gene expression. By binding to 3′-untranslated regions (UTRs) in target mRNAs, miRNAs regulate post-transcriptional gene expression by causing translational repression or mRNA degradation [10,11]. Through these mechanisms, miRNAs are involved in the regulation of cell growth, cell proliferation, apoptosis, and cell differentiation [12]. In addition, miRNAs play essential roles in the cardiovascular system by regulating heart and blood vessel development as well as cardiovascular diseases [13]. Recently, several miRNAs were found to regulate CAD development [14,15].

The *PAI-1* gene, which encodes a member of the serine protease inhibitor superfamily, is located on chromosome 7. *PAI-1* is produced by endothelial cells, platelets, and other cell types and is associated with several disease conditions including diabetes, hypertension, metabolic syndrome (MetS), and obesity. When plasminogen is converted to plasmin, the fibrinolytic system is initiated [16]. When plasmin is activated by tissue plasminogen activator (tPA) and urokinase plasminogen activator (uPA), it causes fibrinolysis, matrix metalloprotease activation, and extracellular matrix degradation [17]. This fibrinolytic system is regulated by *PAI-1*. Studies have shown that polymorphisms in *PAI-1* are associated with CAD [18].

The above data suggests a role for both *PAI-1* and miRNAs in CAD. Based on these associations, we wondered how expression-altering miRNAs that bind to the 3′-UTR of *PAI-1* impact the risk for CAD and if there are any associations between polymorphisms in these miRNA genes and other CAD risk factors. To answer these questions, we designed a genetic epidemiological study to test for associations between CAD risk in the Korean population and five polymorphisms of miRNAs binding to the 3′-UTR of the *PAI-1* gene: *miR-30c* rs928508 A > G, *miR-143* rs41291957 G > A, *miR-143* rs4705342 T > C, *miR-145* rs353291 T > C, and *miR-181a2* rs10760371 T > G.

## 2. Results

### 2.1. Characteristics of the Study Participants

Table 1 displays the clinical characteristics of the 483 patients with CAD and the 400 control participants. The comparison between patients and controls did not reveal any significant differences in age or sex (*p* = 0.511 and *p* = 0.124, respectively). The average body mass index (BMI) of the patients (mean ± standard deviation [SD] = 24.92 ± 3.48) was significantly higher than that of the controls (24.33 ± 3.39). Diabetes mellitus, hypertension, and MetS, which are major risk factors for CAD, were more prevalent among the patients than among the controls (*p* < 0.05 for each). There were no statistically significant differences in hyperlipidemia or smoking status between the two groups. Fasting blood sugar, high-density lipoprotein cholesterol (HDL-cholesterol), Total cholesterol, and folate and creatinine levels were significantly different between the patients and controls (*p* < 0.05 for each). There were no differences in the levels of low-density lipoprotein cholesterol (LDL-cholesterol) (*p* = 0.072), triglycerides (*p* = 0.074), homocysteine (*p* = 0.861), or vitamin B12 (*p* = 0.314) between the patients and the controls.

### 2.2. Genotype Frequencies of miRNA SNPs

To assess potential correlations between CAD risk and the five polymorphisms in miRNAs known to target the *PAI-1* 3′-UTR, we measured the genotype frequencies of each polymorphism in the patient and the control cohorts (Table 2). We found that the genotype frequencies of the specified SNPs in both cohorts conformed to the Hardy–Weinberg equilibrium (HWE; *p* > 0.05), suggesting that the population distribution was consistent with the principles of HWE. For *miR-30c* rs928508, in an adjusted statistical analysis considering age, sex, hypertension, and diabetes, there was a significant difference in the GG genotype [adjusted odds ratio (AOR) = 1.636, *p* = 0.026] and the recessive model (AOR = 1.527, *p* = 0.037) between the patients and the controls.

### 2.3. Genotype Combination Analysis

We performed a genotype combination analysis to look for the effects of combined miRNA genotypes on CAD risk (Table 3). We found that CAD risk was increased when the homozygous mutant genotype of *miR-30c* rs928508 was combined with the homozygous wild-type genotypes of *miR-143* rs41291957 (GG/GG, AOR = 2.052), *miR-143* rs4705342 (GG/TT, AOR = 1.932), or *miR-181a2* rs10760371 (GG/TT, AOR = 2.890). Conversely, the CAD risk was reduced with certain combinations of genotypes of *miR-143* rs41291957 and *miR-143* rs4705342 (GG/TC, AOR = 0.517; GA/TT, AOR = 0.253; AA/TC, AOR = 0.360). None of the other genotype combinations were associated with CAD risk (Table S1).

### 2.4. Allele Combination Analysis

We performed an allele combination analysis to identify combinations of alleles at the five miRNA polymorphisms that were associated with CAD risk (Table 4). The combination of the mutant allele (G) of *miR-30c* rs928508 and the wild-type allele (T) of *miR-143* rs4705342 was associated with increased CAD risk (AOR = 1.307, 95% CI = 1.034–1.651, *p* = 0.025). These two alleles also increased the CAD risk in three-allele and four-allele combinations with the wild-type alleles of *miR-143* rs41291957 (G), *miR-145* rs353291 (T), and *miR-181a2* rs10760371 (T).

On the other hand, the combination of the wild-type allele (G) of *miR-143* rs41291957 and the mutant allele (C) of *miR-143* rs4705342 was associated with decreased CAD risk (AOR = 0.519, 95% CI = 0.337–0.800, *p* = 0.003). These two alleles also reduced the CAD risk in three-allele and four-allele combinations with the mutant allele of *miR-145* rs353291 (C) and the wild-type allele of *miR-181a2* rs10760371 (T) (Table 5). The mutant allele (A) of *miR-143* rs41291957 and the wild-type allele (T) of *miR-143* rs4705342 were also associated with decreased CAD risk in a two-allele combination (AOR = 0.249, 95% CI = 0.121–0.513, *p* < 0.0001) and in three-allele and four-allele combinations with the wild-type alleles of *miR-145* rs353291 (T) and *miR-181a2* rs10760371 (T).

### 2.5. Synergistic Effects of miRNA Polymorphisms and Clinical Parameters

**Figure 1:**
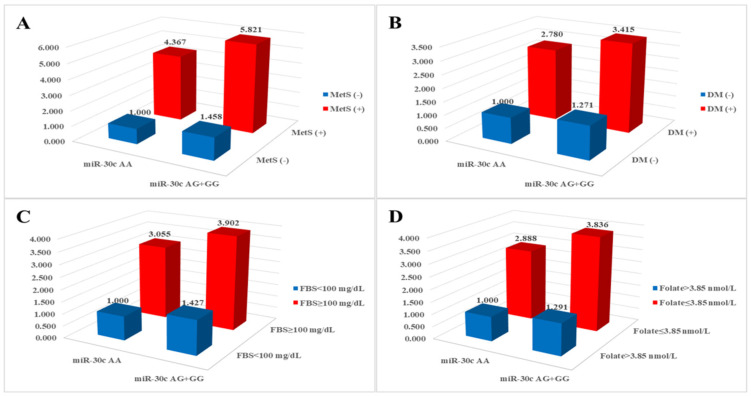
Analysis of synergistic effects on coronary artery disease risk between *miR-30c* rs928508 and (**A**) metabolic syndrome (MetS), (**B**) diabetes mellitus (DM), (**C**) fasting blood sugar (FBS), and (**D**) folate.

We investigated the combined effects of miRNA polymorphisms and clinical parameters and found that various clinical parameters exhibited synergistic effects with miRNA polymorphisms (Table S2). Specifically, MetS-related clinical parameters in conjunction with *miR-30c* rs928508 were significantly associated with an elevated risk of CAD, as illustrated in Figure 1. The AOR for individuals with the *miR-30c* rs928508 AG + GG genotypes and diabetes mellitus, fasting blood sugar ≥ 100 mg/dL, and folate ≤ 3.85 nmol/L was 3.415 (*p* < 0.0001), 3.902 (*p* < 0.0001), and 3.836 (*p* < 0.0001), respectively.

## 3. Discussion

We evaluated associations between CAD risk and five polymorphisms in miRNAs targeting the 3′-UTR of *PAI-1*. Associations have been reported between these five polymorphisms and other diseases, including non-small-cell lung cancer (*miR-30c* rs928508) [19], colorectal cancer (*miR-143* rs41291957) [20], ischemic stroke (*miR-143* rs4705342) [21], atherosclerosis (*miR-145* rs353291) [22], and methamphetamine addiction (*miR-181a2* rs10760371) [23]. However, only *miR-143* rs41291957 was previously investigated in relation to CAD [24]. Furthermore, although these SNPs have been studied in many countries, they have not been analyzed in Korea. Thus, to our knowledge, our study represents the first attempt to elucidate the effect of these five miRNA SNPs on the prevalence of CAD in Koreans.

In a prior study, *miR-34a* was shown to bind to the 3′UTR of *PAI-1* and regulate *PAI-1* expression, with *miR-34a* overexpression leading to reduced expression of *PAI-1* [25]. Similarly, *miR-143*, another miRNA investigated in this study, was reported to bind to the 3′UTR of *PAI-1* and inhibit its expression [26]. Thus, published findings indicate that miRNA binding to *PAI-1* lowers the expression level of this gene. *PAI-1* encodes a protein that inhibits tPA and uPA, disrupts the fibrinolytic system, and contributes to the pathogenesis of cardiovascular disease. Accordingly, elevated expression of *PAI-1* leads to an increased risk for CAD [27]. Based on the above, we predict that binding of miRNA to the *PAI-1* 3′UTR reduces *PAI-1* expression and lowers the risk for CAD. In our study, CAD risk was significantly increased for those with the GG genotype of *miR-30c* rs928508 (Table 2). This observation suggests that individuals with GG genotype have an elevated incidence of CAD, resulting from reduced expression of *miR-30c* and increased expression of *PAI-1* relative to those with the AA genotype.

CAD is a complex condition influenced by a variety of environmental and genetic risk factors, including *PAI-1* and its associated miRNAs [28,29,30,31]. Several studies have indicated a strong association between components of MetS and atherosclerotic diseases, including CAD [32,33]. Based on the ATP III criteria, a diagnosis of MetS is established when an individual exhibits three or more of the following five criteria: blood pressure ≥ 130/85 mmHg, waist circumference > 102 cm in males or >88 cm in females, fasting blood sugar ≥ 110 mg/dL, plasma triglyceride concentration ≥ 150 mg/dL, and plasma HDL-cholesterol concentration < 40 mg/dL in males or <50 mg/dL in females [34]. Diabetes mellitus, another clinical characteristic, is also associated with CAD [35,36]. On the other hand, genetic predisposition to elevated folate levels was linked to a reduced likelihood of developing CAD [37]. Combinations of *miR-30c* rs928508 genotypes and risk factors associated with MetS demonstrated synergistic elevation of risk for CAD, as illustrated in Figure 1. Subgroups characterized by the *miR-30c* AG + GG genotypes and the presence of hypertension, diabetes mellitus, hyperlipidemia, BMI ≥ 25 kg/m^2^, and HDL < 40 mg/dL (males) or <50 mg/dL (females) displayed significantly increased AORs (2.246, 3.415, 1.675, 1.887, and 2.008, respectively) compared with subgroups with the *miR-30c* AA genotype without MetS-related conditions (Table S2). Consistent with our findings, MetS was also shown to be an important risk factor for CAD in patients from other ethnic groups [38,39].

Our study has several limitations. First, although the regulatory effect of miRNA on PAI-1 is known [25,26], the specific mechanisms through which the five SNPs impact the development of CAD are not fully understood. Therefore, further in vitro and in vivo studies are needed to validate the effects of the SNPs on CAD risk and enhance our understanding for future applications. Second, our study sample was limited to the Korean population, which restricts the generalizability of our findings. We further note that the frequencies of these polymorphisms in the Korean population differ slightly from those in other populations, making it difficult to extend our conclusions to a global context. Consequently, it is crucial to expand our research to encompass a more diverse range of patient samples. Third, there is a dearth of information regarding other environmental risk factors for CAD.

In summary, we evaluated whether five polymorphisms of *PAI-1*-related miRNAs affect susceptibility to CAD in the Korean population. We found that *miR-30c* rs928508 was associated with CAD risk. In combination analyses, some combinations of *miR-30c* rs928508, *miR-143* rs41291957, and *miR-143* rs4705342 alleles and genotypes were also associated with increased or decreased CAD risk. Moreover, these three polymorphisms displayed synergistic effects on CAD risk when combined with clinical conditions that independently increase the risk of CAD. These findings can be used to identify new CAD prognostic biomarkers using *miR-30c* rs928508, *miR-143* rs41291957, and *miR-143* rs4705342 combined with other miRNA polymorphisms and various clinical factors.

## 4. Materials and Methods

### 4.1. Study Participants

Blood samples were obtained from 483 patients with CAD [age, mean ± standard deviation (SD) = 61.07 ± 11.46 years] and 400 age- and sex-matched healthy control participants (age, mean ± SD = 60.56 ± 11.70 years). All participants were recruited from the Department of Cardiology of CHA Bundang Medical Center, CHA University, in Seongnam, South Korea, between 2014 and 2016. All participants provided written informed consent for the study, which was approved by the Institutional Review Board of CHA Bundang Medical Center (IRB number: 2013-10-114). All study procedures adhered to the principles outlined in the Declaration of Helsinki.

The patients included in this study exhibited coronary artery stenosis of over 50% in at least one of the primary coronary arteries or their significant branches, as verified by coronary angiography. Patients with a history of cardiac arrest and a life expectancy of less than one year were excluded to mitigate the confounding effects of diverse medical interventions on blood testing. Diagnoses were established via coronary angiography and were corroborated by at least one proficient cardiologist. The 400 control participants were seen at the Department of Cardiology at the CHA Bundang Medical Center for a thorough health assessment, which included biochemical testing and cardiological examination. Individuals with a history of angina or myocardial infarction, as well as those exhibiting T wave inversion on electrocardiography, were excluded from the control cohort.

Hypertension was characterized by systolic pressure ≥ 130 mmHg and diastolic pressure ≥ 80 mmHg and was considered present in individuals using anti-hypertensive medications [40]. Diabetes mellitus was defined as a fasting plasma glucose level ≥ 110 mg/dL and was considered present in individuals taking medications for diabetes. Hyperlipidemia was defined as fasting serum total cholesterol (TC) ≥ 150 mg/dL or a history of treatment with anti-hyperlipidemic agents. Smoking status referred to individuals currently engaged in smoking [18].

### 4.2. Blood Biochemical Analyses

Blood (2 mL) was obtained in tubes containing anticoagulant after a 12 h fasting period. To isolate plasma from whole blood, the samples were centrifuged at 1000× *g* for 15 min. Plasma concentrations of homocysteine and folate were measured using the IMx fluorescence polarizing immunoassay (Abbott Laboratories, Abbott Park, IL, USA) and a radioimmunoassay kit (ACS:180; Bayer, Tarrytown, NY, USA), respectively. The levels of total cholesterol, triglycerides, HDL-cholesterol, and LDL-cholesterol were determined by colorimetric enzymatic methods using commercial reagent sets (TBA 200FR NEO, Toshiba Medical Systems, Otawara, Japan).

### 4.3. SNP Selection

To identify miRNAs that bind to *PAI-1* mRNA, an online search was performed using TargetScan (http://www.targetscan.org, accessed on 5 September 2023) (Figure S1) and TarBase v8 databases (DIANA tools—TarBase v8 (uth.gr), accessed on 5 September 2023) (Figure S2). The targeted mRNA sequences, primarily located in the 3′-UTR, were acquired from the National Center for Biotechnology Information (NCBI; www.ncbi.nlm.nih.gov/, accessed on 5 September 2023). We first selected overlapping SNPs from the results of an online search. We then searched the SNPs in NCBI and selected only those found in East Asian populations and with an alternative allele frequency of ≥0.05.

### 4.4. Genetic Analyses

DNA was isolated from white blood cells in peripheral blood using the G-dex II Genomic DNA Extraction kit (iNtRON Biotechnology, Inc., Seongnam, Republic of Korea), following the guidelines provided by the manufacturer. After extraction, the quantity (A260) and quality (A260/A280 ratio) of genomic DNA were promptly evaluated with a NanoDrop 2000 (Thermo Fisher Scientific, Waltham, MA, USA). Genomic DNA purity was further confirmed by analyzing the patterns of DNA fragments obtained from agarose gel electrophoresis.

*miR-30c* rs928508 A > G, *miR-143* rs41291957 G > A, *miR-143* rs4705342 T > C, *miR-145* rs353291 T > C, and *miR-181a2* rs10760371 T > G were genotyped using a TaqMan™ SNP Genotyping Assay Kit (Thermo Fisher Scientific, Inc., Waltham, MA, USA) with a Rotor-Gene 6000 Real-Time PCR System (QIAGEN, Hilden, Germany). A solution was prepared for real-time PCR by combining 1 µL genomic DNA (100 ng/µL), 7.5 µL TaqMan Genotyping Master Mix (Applied Biosystems, Waltham, MA, USA), 0.75 µL TaqMan SNP Genotyping Assay (Applied Biosystems), and distilled water to achieve a final volume of 15 µL. The experimental procedure included appropriate negative controls. The thermal cycling conditions for this experiment were as follows: initial denaturation at 95 °C for 10 min, followed by 40 cycles of denaturation at 95 °C for 15 s and annealing at 60 °C for 1 min [41]. To confirm the genotypes, 30% of the samples for each polymorphism were randomly selected and subjected to base sequence analysis using an ABI 3730xl DNA Analyzer (Applied Biosystems, Foster City, CA, USA). The sequencing results were 100% consistent with the genotyping results. Primer sequences for the five SNPs are listed in Table S3.

### 4.5. Statistical Analysis

To compare clinical characteristics between the controls and the patients with CAD, Chi-square tests and Student’s *t*-tests were used for categorical data and continuous data, respectively. Logistic regression was used to estimate associations between the polymorphisms and CAD risk. The AORs were adjusted by age, sex, hypertension, diabetes mellitus, hyperlipidemia, and smoking status. Genotyping was performed by treating the most common homozygous genotype as the dominant model and the less common homozygous genotype as the recessive model. To minimize false positives in the results, a false discovery rate (FDR) correction was applied to the *p*-values using the formula q = *p* * (n/k), where n represents the total number of *p*-values and k indicates the rank of the *p*-values when arranged from smallest to largest [42]. One-way analysis of variance was performed to investigate the differences in various clinical factors depending on the genotypes of the polymorphisms. Statistical significance was accepted at the *p* < 0.05 level. The allele frequencies obtained from each cohort were calculated to assess the adherence or departure from HWE. Analyses were performed using GraphPad Prism 4.0 (GraphPad Software Inc., San Diego, CA, USA) and Medcalc version 12.7.1.0 (Medcalc Software, Mariakerke, Belgium). Haplotypes for multiple loci were estimated using the expectation–maximization algorithm with SNPAlyze (version 5.1; DYNACOM Co, Ltd., Yokohama, Japan).

## Figures and Tables

**Table 1:** Baseline characteristics between CAD and controls.

Characteristic	Controls (n = 400)	CAD Patients (n = 483)	*p*
Age (years, mean ± SD)	60.56 ± 11.70	61.07 ± 11.46	0.511
Male (n, %)	167 (41.8)	243 (50.3)	0.124
BMI (kg/m^2^, mean ± SD)	24.33 ± 3.39	24.92 ± 3.48	0.026
Hypertension (n, %)	153 (38.3)	257 (53.2)	0.007
Diabetes mellitus (%)	48 (12.0)	128 (26.5)	<0.0001
Hyperlipidemia (n, %)	91 (22.8)	126 (26.1)	0.372
Smoking (n, %)	129 (32.3)	161 (33.3)	0.808
Metabolic syndrome (n, %)	107 (26.8)	284 (58.8)	<0.0001
Fasting blood sugar (mg/dL, mean ± SD)	113.09 ± 35.24	141.76 ± 63.25	<0.0001
HDL-C (mg/dL, mean ± SD)	46.77 ± 13.30	43.67 ± 10.98	0.013
LDL-C (mg/dL, mean ± SD)	117.81 ± 40.65	111.37 ± 39.43	0.072
Total cholesterol (mg/dL, mean ± SD)	192.48 ± 37.19	184.35 ± 45.14	0.001
Triglyceride (mg/dL, mean ± SD)	142.96 ± 88.36	154.08 ± 93.38	0.074
Homocysteine (μmol/L, mean ± SD)	9.73 ± 4.08	9.99 ± 4.78	0.861
Vitamin B12 (pg/mL, mean ± SD)	693.19 ± 291.81	657.92 ± 315.23	0.314
Folate (nmol/L, mean ± SD)	8.86 ± 7.85	8.07 ± 6.95	0.001
Creatinine (mg/dL, mean ± SD)	0.94 ± 0.22	1.31 ± 4.67	<0.0001

Note: CAD, coronary artery disease; SD, standard deviation; BMI, body mass index; HDL-C, high-density lipoprotein cholesterol; LDL-C, low-density lipoprotein cholesterol. *p*-value was calculated using Chi-square test for categorical variables and Mann–Whitney test for continuous variables.

**Table 2:** Genotype frequencies of *PAI-1*-related miRNA polymorphisms in CAD and controls.

Genotypes	Controls	CAD	AOR (95% CI)	*p*	FDR-*p*
	(n = 400)	(n = 483)			
*miR-30c* rs928508					
AA	157 (39.3)	160 (33.1)			
AG	196 (49.0)	235 (48.7)	1.166 (0.862–1.576)	0.319	0.709
GG	47 (11.8)	88 (18.2)	1.636 (1.061–2.523)	0.026	0.516
Dominant (AA vs. AG + GG)			1.257 (0.943–1.674)	0.119	0.790
Recessive (AA + AG vs. GG)			1.527 (1.026–2.273)	0.037	0.369
HWE-P	0.229	0.916			
*miR-143* rs41291957					
GG	197 (49.3)	246 (50.9)			
GA	161 (40.3)	193 (40.0)	0.879 (0.655–1.179)	0.388	0.646
AA	42 (10.5)	44 (9.1)	0.778 (0.478–1.267)	0.313	0.783
Dominant (GG vs. GA + AA)			0.864 (0.655–1.138)	0.298	0.850
Recessive (GG + GA vs. AA)			0.825 (0.519–1.313)	0.417	0.642
HWE-P	0.291	0.49			
*miR-143* rs4705342					
TT	175 (43.8)	224 (46.4)			
TC	181 (45.3)	210 (43.5)	0.833 (0.622–1.114)	0.218	0.872
CC	44 (11.0)	49 (10.1)	0.790 (0.490–1.273)	0.333	0.666
Dominant (TT vs. TC + CC)			0.823 (0.623–1.086)	0.168	0.839
Recessive (TT + TC vs. CC)			0.867 (0.554–1.359)	0.534	0.712
HWE-P	0.784	0.983			
*miR-145* rs353291					
TT	129 (32.3)	170 (35.2)			
TC	202 (50.5)	233 (48.2)	0.885 (0.651–1.203)	0.434	0.620
CC	69 (17.3)	80 (16.6)	1.044 (0.687–1.587)	0.839	0.839
Dominant (TT vs. TC + CC)			0.918 (0.686–1.230)	0.567	0.709
Recessive (TT + TC vs. CC)			1.077 (0.746–1.555)	0.693	0.770
HWE-P	0.506	0.991			
*miR-181a2* rs10760371					
TT	117 (29.3)	147 (30.4)			
TG	198 (49.5)	246 (50.9)	0.966 (0.702–1.330)	0.833	0.877
GG	85 (21.3)	90 (18.6)	0.822 (0.550–1.228)	0.339	0.616
Dominant (TT vs. TG + GG)			0.917 (0.678–1.241)	0.575	0.677
Recessive (TT + TG vs. GG)			0.826 (0.585–1.168)	0.280	0.934
HWE-P	0.942	0.468			

Note: PAI-1, plasminogen activator inhibitor-1; CAD, coronary artery disease; FDR, false discovery rate; CI, confidence interval; AOR, adjusted odds ratio; HWE, Hardy–Weinberg equilibrium. *p*-value was calculated using logistic regression. AOR was adjusted by age, sex, hypertension, diabetes mellitus, hyperlipidemia, and smoking status.

**Table 3:** Genotype combination frequencies of *PAI-1*-related miRNA polymorphisms in CAD patients and controls.

Combination	Controls (n = 400)	CAD (n = 483)	AOR (95% CI)	*p*
*miR-30c* rs928508 A > G/*miR-143* rs41291957 G > A				
AA/GG	83 (20.8)	81 (16.8)	1.000 (reference)	
AA/GA	53 (13.3)	63 (13.0)	1.140 (0.695–1.872)	0.604
AA/AA	21 (5.3)	16 (3.3)	0.712 (0.334–1.518)	0.379
AG/GG	92 (23.0)	115 (23.8)	1.270 (0.824–1.956)	0.279
AG/GA	85 (21.3)	97 (20.1)	1.062 (0.686–1.644)	0.788
AG/AA	19 (4.8)	23 (4.8)	1.187 (0.585–2.410)	0.635
GG/GG	22 (5.5)	50 (10.4)	2.052 (1.107–3.807)	0.023
GG/GA	23 (5.8)	33 (6.8)	1.254 (0.654–2.405)	0.496
GG/AA	2 (0.5)	5 (1.0)	2.369 (0.407–3.794)	0.337
*miR-30c* rs928508 A > G/*miR-143* rs4705342 T > C				
AA/TT	75 (18.8)	74 (15.3)	1.000 (reference)	
AA/TC	57 (14.3)	69 (14.3)	1.096 (0.666–1.802)	0.719
AA/CC	25 (6.3)	17 (3.5)	0.672 (0.328–1.378)	0.278
AG/TT	78 (19.5)	105 (21.7)	1.284 (0.816–2.019)	0.280
AG/TC	102 (25.5)	103 (21.3)	0.937 (0.605–1.453)	0.771
AG/CC	16 (4.0)	27 (5.6)	1.558 (0.757–3.205)	0.228
GG/TT	22 (5.5)	45 (9.3)	1.932 (1.030–3.624)	0.040
GG/TC	22 (5.5)	38 (7.9)	1.591 (0.839–3.017)	0.155
GG/CC	3 (0.8)	5 (1.0)	1.017 (0.202–5.110)	0.984
*miR-30c* rs928508 A > G/*miR-181a2* rs10760371 T > G				
AA/TT	53 (13.3)	52 (10.8)	1.000 (reference)	
AA/TG	74 (18.5)	79 (16.4)	1.098 (0.654–1.842)	0.724
AA/GG	30 (7.5)	29 (6.0)	0.935 (0.469–1.866)	0.849
AG/TT	52 (13.0)	59 (12.2)	1.319 (0.750–2.319)	0.337
AG/TG	99 (24.8)	126 (26.1)	1.264 (0.774–2.061)	0.349
AG/GG	45 (11.3)	50 (10.4)	1.152 (0.644–2.060)	0.634
GG/TT	12 (3.0)	36 (7.5)	2.890 (1.314–6.354)	0.008
GG/TG	25 (6.3)	41 (8.5)	1.491 (0.755–2.947)	0.250
GG/GG	10 (2.5)	11 (2.3)	0.965 (0.347–2.685)	0.946
*miR-143* rs41291957 G > A/*miR-143* rs4705342 T > C				
GG/TT	160 (40.0)	220 (45.5)	1.000 (reference)	
GG/TC	32 (8.0)	23 (4.8)	0.517 (0.283–0.944)	0.032
GG/CC	5 (1.3)	3 (0.6)	0.328 (0.068–1.578)	0.164
GA/TT	14 (3.5)	4 (0.8)	0.253 (0.079–0.812)	0.021
GA/TC	136 (34.0)	181 (37.5)	0.879 (0.643–1.201)	0.418
GA/CC	11 (2.8)	8 (1.7)	0.513 (0.192–1.368)	0.182
AA/TT	1 (0.3)	0 (0.0)	N/A	N/A
AA/TC	13 (3.3)	6 (1.2)	0.360 (0.130–0.996)	0.049
AA/CC	28 (7.0)	38 (7.9)	0.910 (0.524–1.582)	0.739

Note: PAI-1, plasminogen activator inhibitor-1; CAD, coronary artery disease; AOR, adjusted odds ratio; CI, confidence interval; N/A, not applicable. *p*-value was calculated using logistic regression. AOR was adjusted by age, sex, hypertension, diabetes mellitus, hyperlipidemia, and smoking status.

**Table 4:** Summary of results from allele combination analysis in CAD patients and controls identifying PAI-1–related miRNA polymorphisms associated with increased CAD risk.

Allele Combinations	Controls (2n = 800)	Cases (2n = 966)	OR (95%CI)	*p*
*miR-30c* rs928508 A > G/*miR-143* rs4705342 T > C				
A-T	332 (41.5)	369 (38.2)	1.000 (reference)	
A-C	178 (22.3)	186 (19.3)	0.940 (0.73–1.211)	0.633
G-T	199 (24.9)	289 (29.9)	1.307 (1.034–1.651)	0.025
G-C	91 (11.4)	122 (12.6)	1.206 (0.885–1.644)	0.235
*miR-30c* rs928508 A > G/*miR-143* rs4705342 T > C/*miR-143* rs41291957 G > A				
A-T-G	312 (39.0)	363 (37.6)	1.000 (reference)	
G-T-G	188 (23.5)	285 (29.5)	1.303 (1.027–1.654)	0.029
*miR-30c* rs928508 A > G/*miR-143* rs4705342 T > C/*miR-145* rs353291 T > C				
A-T-T	170 (21.1)	190 (19.7)	1.000 (reference)	
G-T-T	95 (11.9)	157 (16.3)	1.470 (1.058–2.042)	0.021
*miR-30c* rs928508 A > G/*miR-143* rs4705342 T > C/*miR-181a2* rs10760371 T > G				
A-T-T	178 (22.3)	193 (19.9)	1.000 (reference)	
G-T-T	97 (12.1)	169 (17.5)	1.615 (1.17–2.23)	0.004
*miR-30c* rs928508 A > G/*miR-143* rs4705342 T > C/*miR-143* rs41291957 G > A/*miR-145* rs353291 T > C				
A-T-G-T	158 (19.9)	186 (19.4)	1.000 (reference)	
G-T-G-T	85 (10.6)	153 (15.8)	1.530 (1.09–2.149)	0.014
*miR-30c* rs928508 A > G/*miR-143* rs4705342 T > C/*miR-143* rs41291957 G > A/*miR-181a2* rs10760371 T > G				
A-T-G-T	164 (20.3)	189 (19.6)	1.000 (reference)	
G-T-G-T	97 (12.1)	163 (16.9)	1.440 (1.038–1.998)	0.029
*miR-30c* rs928508 A > G/*miR-143* rs4705342 T > C/*miR-145* rs353291 T > C/*miR-181a2* rs10760371 T > G				
A-T-T-T	92 (11.4)	95 (9.9)	1.000 (reference)	
G-T-T-T	52 (6.5)	91 (9.4)	1.659 (1.063–2.59)	0.026

Note: PAI-1, plasminogen activator inhibitor-1; CAD, coronary artery disease; OR, odds ratio; CI, confidence interval. *p*-value was calculated using Chi-square test.

**Table 5:** Summary of results from allele combinations analysis in CAD patients and controls identifying PAI-1–related miRNA polymorphisms associated with decreased CAD risk.

Allele (2n = 800) Cases	Controls (2n = 800)	Cases (2n = 966)	OR (95% CI)	*p*
*miR-143* rs41291957 G > A/*miR-143* rs4705342 T > C				
G-T	500 (62.5)	648 (67.1)	1.000 (reference)	
G-C	55 (6.9)	37 (3.8)	0.519 (0.337–0.800)	0.003
A-T	31 (3.9)	10 (1.0)	0.249 (0.121–0.513)	<0.0001
A-C	214 (26.8)	271 (28.1)	0.977 (0.789–1.21)	0.832
*miR-143* rs41291957 G > A/*miR-143* rs4705342 T > C/*miR-145* rs353291 T > C				
G-T-T	245 (30.5)	340 (35.1)	1.000 (reference)	
G-C-C	26 (3.3)	12 (1.2)	0.332 (0.164–0.671)	0.001
A-T-T	18 (2.3)	7 (0.7)	0.280 (0.115–0.681)	0.003
*miR-143* rs41291957 G > A/*miR-143* rs4705342 T > C/*miR-181a2* rs10760371 T > G				
G-T-T	258 (32.3)	351 (36.5)	1.000 (reference)	
G-C-T	40 (5.0)	21 (2.2)	0.384 (0.221–0.667)	0.001
A-T-T	16 (2.0)	8 (0.8)	0.365 (0.154–0.867)	0.018
*miR-143* rs41291957 G > A/*miR-143* rs4705342 T > C/*miR-145* rs353291 T > C/*miR-181a2* rs10760371 T > G				
G-T-T-T	132 (16.3)	182 (18.8)	1.000 (reference)	
G-C-C-T	22 (2.8)	11 (1.1)	0.357 (0.167–0.762)	0.006
A-T-T-T	12 (1.5)	3 (0.3)	0.179 (0.049–0.646)	0.006

Note: PAI-1, plasminogen activator inhibitor-1; CAD, coronary artery disease; OR, odds ratio; CI, confidence interval. *p*-value was calculated using Chi-square test.

## Data Availability

The data presented in this study can be made available upon request from the corresponding author.

## Supplementary Materials

The following supporting information can be downloaded at: https://www.mdpi.com/article/10.3390/ijms252111528/s1.

## References

[1] Park H.S., Kim I.J., Kim E.G., Ryu C.S., Lee J.Y., Ko E.J., Park H.W., Sung J.H., Kim N.K. (2020). A study of associations between CUBN, HNF1A, and LIPC gene polymorphisms and coronary artery disease. Sci. Rep..

[2] MacMahon S., Peto R., Cutler J., Collins R., Sorlie P., Neaton J., Abbott R., Godwin J., Dyer A., Stamler J. (1990). Blood pressure, stroke, and coronary heart disease. Part 1, Prolonged differences in blood pressure: Prospective observational studies corrected for the regression dilution bias. Lancet.

[3] Mulcahy R. (1990). The health benefits of smoking cessation. Ir. Med. J..

[4] Stamler J., Vaccaro O., Neaton J.D., Wentworth D. (1993). Diabetes, other risk factors, and 12-yr cardiovascular mortality for men screened in the Multiple Risk Factor Intervention Trial. Diabetes Care.

[5] Verschuren W.M., Jacobs D.R., Bloemberg B.P., Kromhout D., Menotti A., Aravanis C., Blackburn H., Buzina R., Dontas A.S., Fidanza F. (1995). Serum total cholesterol and long-term coronary heart disease mortality in different cultures. Twenty-five-year follow-up of the seven countries study. JAMA.

[6] Bouzidi N., Hassine M., Fodha H., Ben Messaoud M., Maatouk F., Gamra H., Ferchichi S. (2020). Association of the methylene-tetrahydrofolate reductase gene rs1801133 C677T variant with serum homocysteine levels, and the severity of coronary artery disease. Sci. Rep..

[7] Kim I.J., Kim S.H., Cha D.H., Lim S.W., Moon J.Y., Kim J.O., Ryu C.S., Park H.S., Sung J.H., Kim N.K. (2019). Association of COX2 -765G>C promoter polymorphism and coronary artery disease in Korean population. Genes Genomics.

[8] Liang Z., Jiang W., Ouyang M., Yang K. (2015). PAI-1 4G/5G polymorphism and coronary artery disease risk: A meta-analysis. Int. J. Clin. Exp. Med..

[9] Lima L.M., Carvalho M., Fonseca Neto C.P., Garcia J.C., Sousa M.O. (2011). PAI-1 4G/5G polymorphism and plasma levels association in patients with coronary artery disease. Arq. Bras. Cardiol..

[10] Huang S.M., Zhao X., Zhao X.M., Wang X.Y., Li S.S., Zhu Y.H. (2014). Biological mechanism analysis of acute renal allograft rejection: Integrated of mRNA and microRNA expression profiles. Int. J. Clin. Exp. Med..

[11] Kayano M., Higaki S., Satoh J.I., Matsumoto K., Matsubara E., Takikawa O., Niida S. (2016). Plasma microRNA biomarker detection for mild cognitive impairment using differential correlation analysis. Biomark. Res..

[12] Sati I., Parhar I. (2021). MicroRNAs Regulate Cell Cycle and Cell Death Pathways in Glioblastoma. Int. J. Mol. Sci..

[13] Small E.M., Frost R.J., Olson E.N. (2010). MicroRNAs add a new dimension to cardiovascular disease. Circulation.

[14] Ghafouri-Fard S., Gholipour M., Taheri M. (2021). Role of MicroRNAs in the Pathogenesis of Coronary Artery Disease. Front. Cardiovasc. Med..

[15] Andiappan R., Govindan R., Ramasamy T., Poomarimuthu M. (2024). Circulating miR-133a-3p and miR-451a as potential biomarkers for diagnosis of coronary artery disease. Acta Cardiol..

[16] Jung R.G., Motazedian P., Ramirez F.D., Simard T., Di Santo P., Visintini S., Faraz M.A., Labinaz A., Jung Y., Hibbert B. (2018). Association between plasminogen activator inhibitor-1 and cardiovascular events: A systematic review and meta-analysis. Thromb. J..

[17] He C.S., Wilhelm S.M., Pentland A.P., Marmer B.L., Grant G.A., Eisen A.Z., Goldberg G.I. (1989). Tissue cooperation in a proteolytic cascade activating human interstitial collagenase. Proc. Natl. Acad. Sci. USA.

[18] Park H.S., Sung J.H., Ryu C.S., Lee J.Y., Ko E.J., Kim I.J., Kim N.K. (2020). The Synergistic Effect of Plasminogen Activator Inhibitor-1 (PAI-1) Polymorphisms and Metabolic Syndrome on Coronary Artery Disease in the Korean Population. J. Pers. Med..

[19] Hu Z., Shu Y., Chen Y., Chen J., Dong J., Liu Y., Pan S., Xu L., Xu J., Wang Y. (2011). Genetic polymorphisms in the precursor MicroRNA flanking region and non-small cell lung cancer survival. Am. J. Respir. Crit. Care Med..

[20] Li L., Pan X., Li Z., Bai P., Jin H., Wang T., Song C., Zhang L., Gao L. (2013). Association between polymorphisms in the promoter region of miR-143/145 and risk of colorectal cancer. Hum. Immunol..

[21] Wei Y.S., Xiang Y., Liao P.H., Wang J.L., Peng Y.F. (2016). An rs4705342 T>C polymorphism in the promoter of miR-143/145 is associated with a decreased risk of ischemic stroke. Sci. Rep..

[22] Lv Y., Yi Y., Jia S., Peng X., Yang H., Guo R. (2020). The miR-145 rs353291 C allele increases susceptibility to atherosclerosis. Front. Biosci..

[23] Sun Q., Zhao Y., Zhang K., Su H., Chen T., Jiang H., Du J., Zhong N., Yu S., Zhao M. (2020). An association study between methamphetamine use disorder with psychosis and polymorphisms in MiRNA. Neurosci. Lett..

[24] Hall I.F., Climent M., Viviani Anselmi C., Papa L., Tragante V., Lambroia L., Farina F.M., Kleber M.E., März W., Biguori C. (2021). rs41291957 controls miR-143 and miR-145 expression and impacts coronary artery disease risk. EMBO Mol. Med..

[25] Zhang Y., Pan Y., Xie C., Zhang Y. (2018). miR-34a exerts as a key regulator in the dedifferentiation of osteosarcoma via PAI-1-Sox2 axis. Cell Death Dis..

[26] Hirahata M., Osaki M., Kanda Y., Sugimoto Y., Yoshioka Y., Kosaka N., Takeshita F., Fujiwara T., Kawai A., Ito H. (2016). PAI-1, a target gene of miR-143, regulates invasion and metastasis by upregulating MMP-13 expression of human osteosarcoma. Cancer Med..

[27] Sillen M., Declerck P.J. (2020). Targeting PAI-1 in Cardiovascular Disease: Structural Insights Into PAI-1 Functionality and Inhibition. Front. Cardiovasc. Med..

[28] Agiannitopoulos K., Samara P., Papadopoulou M., Efthymiadou A., Papadopoulou E., Tsaousis G.N., Mertzanos G., Babalis D., Lamnissou K. (2021). miRNA polymorphisms and risk of premature coronary artery disease. Hellenic J. Cardiol..

[29] Malakar A.K., Choudhury D., Halder B., Paul P., Uddin A., Chakraborty S. (2019). A review on coronary artery disease, its risk factors, and therapeutics. J. Cell Physiol..

[30] Sarecka B., Zak I., Krauze J. (2008). Synergistic effects of the polymorphisms in the PAI-1 and IL-6 genes with smoking in determining their associated risk with coronary artery disease. Clin. Biochem..

[31] Wang T., Yuan L., Chen Y., Wang J., Li N., Zhou H. (2023). Expression profiles and bioinformatic analysis of microRNAs in myocardium of diabetic cardiomyopathy mice. Genes Genomics.

[32] Zidi W., Allal-Elasmi M., Zayani Y., Zaroui A., Guizani I., Feki M., Mourali M.S., Mechmeche R., Kaabachi N. (2015). Metabolic Syndrome, Independent Predictor for Coronary Artery Disease. Clin. Lab..

[33] Khaliq A., Johnson B.D., Anderson R.D., Bavry A.A., Cooper-DeHoff R.M., Handberg E.M., Bairey Merz C.N., Nicholls S.J., Nissen S., Pepine C.J. (2015). Relationships between components of metabolic syndrome and coronary intravascular ultrasound atherosclerosis measures in women without obstructive coronary artery disease: The NHLBI-Sponsored Women’s Ischemia Syndrome Evaluation Study. Cardiovasc. Endocrinol..

[34] (2002). Third Report of the National Cholesterol Education Program (NCEP) Expert Panel on Detection, Evaluation, and Treatment of High Blood Cholesterol in Adults (Adult Treatment Panel III) final report. Circulation.

[35] Aronson D., Edelman E.R. (2014). Coronary artery disease and diabetes mellitus. Cardiol. Clin..

[36] Einarson T.R., Acs A., Ludwig C., Panton U.H. (2018). Prevalence of cardiovascular disease in type 2 diabetes: A systematic literature review of scientific evidence from across the world in 2007–2017. Cardiovasc. Diabetol..

[37] Yuan S., Mason A.M., Carter P., Burgess S., Larsson S.C. (2021). Homocysteine, B vitamins, and cardiovascular disease: A Mendelian randomization study. BMC Med..

[38] Montazerifar F., Bolouri A., Mahmoudi Mozaffar M., Karajibani M. (2016). The Prevalence of Metabolic Syndrome in Coronary Artery Disease Patients. Cardiol. Res..

[39] Alshammary A.F., Alharbi K.K., Alshehri N.J., Vennu V., Ali Khan I. (2021). Metabolic Syndrome and Coronary Artery Disease Risk: A Meta-Analysis of Observational Studies. Int. J. Environ. Res. Public. Health.

[40] Iqbal A.M., Jamal S.F. (2024). Essential Hypertension. StatPearls.

[41] Kim J.H., Park H.S., Lee J.Y., Ko E.J., Kim Y.R., Cho H.Y., Lee W.S., Ahn E.H., Kim N.K. (2022). Association Study between Mucin 4 (MUC4) Polymorphisms and Idiopathic Recurrent Pregnancy Loss in a Korean Population. Genes..

[42] Li Q., Zhang C., Cheng Y., Yang X., Chen W., He K., Chen M. (2023). IL1RL1 polymorphisms rs12479210 and rs1420101 are associated with increased lung cancer risk in the Chinese Han population. Front. Genet..

