# Supplementary material for: Genetic Associations of Plasminogen Activator Inhibitor-1-Related miRNA Variants with Coronary Artery Disease
Source: Int J Mol Sci. 2024 Oct 27;25(21):11528. doi: 10.3390/ijms252111528 (PMC11546797; doi:10.3390/ijms252111528)
Supplement: Supplementary file 1 [file ijms-25-11528-s001.zip › ijms-3244659-supplementary.pdf]

Table S1. Genotype combination frequencies of *PAI-1* related miRNA polymorphisms in CAD patients and controls.

Combination	Controls (n=400)	CAD (n=483)	AOR (95% CI)	*P*
miR-30c rs928508 A>G/miR-145 rs353291 T>C				
AA/TT	54 (13.5)	53 (11.0)	1.000 (reference)	
AA/TC	74 (18.5)	84 (17.4)	1.254 (0.751-2.093)	0.387
AA/CC	29 (7.3)	23 (4.8)	1.021 (0.497-2.098)	0.954
AG/TT	66 (16.5)	96 (19.9)	1.595 (0.946-2.689)	0.080
AG/TC	99 (24.8)	98 (20.3)	1.030 (0.635-1.672)	0.905
AG/CC	31 (7.8)	41 (8.5)	1.644 (0.864-3.130)	0.130
GG/TT	9 (2.3)	21 (4.3)	2.188 (0.869-5.509)	0.096
GG/TC	29 (7.3)	51 (10.6)	1.807 (0.974-3.353)	0.061
GG/CC	9 (2.3)	16 (3.3)	1.805 (0.717-4.549)	0.210
miR-143 rs41291957 G>A/miR-145 rs353291 T>C				
GG/TT	49 (12.3)	71 (14.7)	1.000 (reference)	
GG/TC	99 (24.8)	122 (25.3)	0.868 (0.542-1.388)	0.554
GG/CC	49 (12.3)	53 (11.0)	0.837 (0.470-1.490)	0.546
GA/TT	57 (14.3)	74 (15.3)	0.828 (0.487-1.407)	0.484
GA/TC	89 (22.3)	92 (19.0)	0.662 (0.406-1.080)	0.099
GA/CC	15 (3.8)	27 (5.6)	1.377 (0.633-2.998)	0.420
AA/TT	23 (5.8)	25 (5.2)	0.774 (0.375-1.598)	0.489
AA/TC	14 (3.5)	19 (3.9)	0.974 (0.427-2.219)	0.950
AA/CC	5 (1.3)	0 (0.0)	N/A	N/A
miR-143 rs41291957 G>A/miR-181a2 rs10760371 T>G				
GG/TT	58 (14.5)	68 (14.1)	1.000 (reference)	
GG/TG	101 (25.3)	135 (28.0)	1.051 (0.664-1.666)	0.831
GG/GG	38 (9.5)	43 (8.9)	0.913 (0.504-1.652)	0.762
GA/TT	43 (10.8)	61 (12.6)	1.092 (0.633-1.883)	0.752
GA/TG	78 (19.5)	92 (19.0)	0.956 (0.590-1.547)	0.853
GA/GG	40 (10.0)	40 (8.3)	0.699 (0.381-1.283)	0.248
AA/TT	16 (4.0)	18 (3.7)	0.841 (0.379-1.869)	0.672
AA/TG	19 (4.8)	19 (3.9)	0.736 (0.343-1.581)	0.432
AA/GG	7 (1.8)	7 (1.4)	0.880 (0.254-3.057)	0.841
miR-143 rs4705342 T>C/miR-145 rs353291 T>C				
TT/TT	48 (12.0)	60 (12.4)	1.000 (reference)	
TT/TC	57 (14.3)	84 (17.4)	1.108 (0.648-1.892)	0.709

TT/CC	24 (6.0)	26 (5.4)	0.938 (0.454-1.937)	0.862
TC/TT	83 (20.8)	115 (23.8)	1.171 (0.715-1.916)	0.531
TC/TC	103 (25.8)	95 (19.7)	0.695 (0.425-1.136)	0.147
TC/CC	16 (4.0)	23 (4.8)	1.064 (0.470-2.406)	0.882
CC/TT	44 (11.0)	49 (10.1)	1.050 (0.574-1.920)	0.875
CC/TC	21 (5.3)	31 (6.4)	1.374 (0.667-2.830)	0.389
CC/CC	4 (1.0)	0 (0.0)	N/A	N/A
miR-143 rs4705342 T>C/miR-181a2 rs10760371 T>G				
TT/TT	50 (12.5)	63 (13.0)	1.000 (reference)	
TT/TG	87 (21.8)	124 (25.7)	1.033 (0.637-1.677)	0.894
TT/GG	38 (9.5)	37 (7.7)	0.706 (0.38-1.313)	0.272
TC/TT	48 (12.0)	64 (13.3)	0.959 (0.554-1.661)	0.882
TC/TG	91 (22.8)	100 (20.7)	0.830 (0.510-1.351)	0.453
TC/GG	42 (10.5)	46 (9.5)	0.730 (0.397-1.339)	0.309
CC/TT	19 (4.8)	20 (4.1)	0.732 (0.336-1.593)	0.431
CC/TG	20 (5.0)	22 (4.6)	0.682 (0.314-1.480)	0.333
CC/GG	5 (1.3)	7 (1.4)	1.461 (0.376-5.686)	0.584
miR-145 rs353291 T>C/miR-181a2 rs10760371 T>G				
TT/TT	47 (11.8)	47 (9.7)	1.000 (reference)	
TT/TG	55 (13.8)	87 (18.0)	1.460 (0.841-2.534)	0.179
TT/GG	27 (6.8)	36 (7.5)	1.323 (0.664-2.634)	0.426
TC/TT	47 (11.8)	74 (15.3)	1.551 (0.888-2.708)	0.123
TC/TG	115 (28.8)	123 (25.5)	1.036 (0.626-1.714)	0.891
TC/GG	40 (10.0)	36 (7.5)	0.836 (0.446-1.567)	0.576
CC/TT	23 (5.8)	26 (5.4)	1.229 (0.586-2.578)	0.585
CC/TG	28 (7.0)	36 (7.5)	1.395 (0.716-2.719)	0.328
CC/GG	18 (4.5)	18 (3.7)	1.091 (0.483-2.463)	0.834

Note: PAI-1, plasminogen activator inhibitor-1; CAD, coronary artery disease; AOR, adjusted odds ratio; CI, confidence interval; N/A, not applicable.

P-value was calculated using logistic regression. AOR was adjusted by age, sex, hypertension, diabetes mellitus, hyperlipidemia, and smoking status.

Table S2. Synergistic effect of *miRNA* polymorphisms with clinical risk factor.

Characteristics	*miR-30c* rs928508 A>G		*miR-143* rs41291957 G>A		*miR-143* rs4705342 T>C		*miR-145* rs353291 T>C		*miR-181a2* rs10760371 T>G	
	AA	AG + GG	GG	GA + AA	TT	TC + CC	TT	TC + CC	TT	TG + GG
	AOR (95% CI)	AOR (95% CI)	AOR (95% CI)	AOR (95% CI)	AOR (95% CI)	AOR (95% CI)	AOR (95% CI)	AOR (95% CI)	AOR (95% CI)	AOR (95% CI)
**Sex**										
male	1.000 (reference)	1.544 (1.016-2.348)	1.000 (reference)	0.890 (0.593-1.336)	1.000 (reference)	0.855 (0.569-1.286)	1.000 (reference)	0.858 (0.557-1.321)	1.000 (reference)	1.077 (0.691-1.680)
female	0.932 (0.542-1.603)	0.836 (0.513-1.363)	0.655 (0.411-1.042)	0.534 (0.336-0.847)	0.676 (0.415-1.101)	0.475 (0.294-0.766)	0.530 (0.301-0.936)	0.438 (0.266-0.721)	0.694 (0.376-1.282)	0.493 (0.295-0.826)
**MetS**										
no	1.000 (reference)	1.458 (0.978-2.175)	1.000 (reference)	0.890 (0.611-1.294)	1.000 (reference)	0.804 (0.553-1.168)	1.000 (reference)	0.870 (0.584-1.297)	1.000 (reference)	0.940 (0.624-1.417)
yes	4.367 (2.564-7.438)	5.821 (3.512-9.649)	4.017 (2.469-6.535)	3.012 (1.927-4.709)	3.851 (2.319-6.396)	3.011 (1.915-4.732)	3.878 (2.386-6.302)	3.346 (1.898-5.899)	5.930 (2.949-1.924)	3.461 (2.141-5.595)
**Hypertension**										
no	1.000 (reference)	0.984 (0.665-1.455)	1.000 (reference)	0.971 (0.666-1.415)	1.000 (reference)	0.753 (0.517-1.099)	1.000 (reference)	0.846 (0.564-1.267)	1.000 (reference)	0.925 (0.608-1.408)
yes	1.331 (0.821-2.159)	2.246 (1.476-3.420)	1.983 (1.314-2.993)	1.598 (1.087-2.350)	1.566 (1.023-2.396)	1.542 (1.042-2.282)	1.490 (0.915-2.427)	1.541 (1.009-2.355)	1.885 (1.119-3.175)	1.598 (1.023-2.498)
**Diabetes mellitus**										
No	1.000 (reference)	1.271 (0.928-1.741)	1.000 (reference)	0.945 (0.697-1.283)	1.000 (reference)	0.870 (0.641-1.182)	1.000 (reference)	0.973 (0.702-1.348)	1.000 (reference)	0.867 (0.624-1.203)
Yes	2.780 (1.433-5.393)	3.415 (2.110-5.526)	3.590 (2.031-6.347)	1.812 (1.094-3.001)	3.409 (1.865-6.231)	1.924 (1.179-3.141)	3.165 (1.700-5.894)	2.259 (1.356-3.766)	1.755 (0.825-3.734)	2.315 (1.44-3.722)
**FBS**										
<100 mg/dL	1.000 (reference)	1.427 (0.792-2.572)	1.000 (reference)	0.799 (0.462-1.383)	1.000 (reference)	0.932 (0.538-1.613)	1.000 (reference)	0.614 (0.353-1.070)	1.000 (reference)	0.814 (0.455-1.457)
≥100 mg/dL	3.055 (1.767-5.283)	3.902 (2.317-6.569)	2.513 (1.608-3.927)	2.335 (1.500-3.632)	2.832 (1.770-4.532)	2.372 (1.501-3.747)	1.831 (1.084-3.093)	2.009 (1.272-3.173)	2.767 (1.548-4.945)	2.362 (1.413-3.950)
**Hyperlipidemia**										
no	1.000 (reference)	1.196 (0.858-1.666)	1.000 (reference)	0.870 (0.632-1.197)	1.000 (reference)	0.851 (0.618-1.171)	1.000 (reference)	0.943 (0.674-1.321)	1.000 (reference)	0.951 (0.667-1.354)
yes	1.115 (0.661-1.879)	1.675 (1.070-2.621)	1.305 (0.807-2.111)	1.108 (0.711-1.728)	1.393 (0.846-2.292)	1.035 (0.670-1.599)	1.359 (0.765-2.413)	1.140 (0.739-1.759)	1.421 (0.815-2.478)	1.224 (0.773-1.938)
**Smoking**										
no	1.000 (reference)	1.363 (0.954-1.947)	1.000 (reference)	0.925 (0.658-1.302)	1.000 (reference)	0.911 (0.647-1.282)	1.000 (reference)	0.888 (0.621-1.272)	1.000 (reference)	0.910 (0.628-1.317)
yes	1.277 (0.727-2.242)	1.267 (0.776-2.070)	1.074 (0.658-1.750)	0.766 (0.469-1.253)	1.167 (0.693-1.967)	0.693 (0.424-1.132)	0.802 (0.442-1.455)	0.674 (0.408-1.113)	0.954 (0.495-1.836)	0.751 (0.455-1.238)
**BMI**										
<25 kg/m2	1.000 (reference)	1.407 (0.916-2.163)	1.000 (reference)	0.781 (0.516-1.183)	1.000 (reference)	0.725 (0.479-1.095)	1.000 (reference)	0.684 (0.436-1.074)	1.000 (reference)	0.946 (0.609-1.469)
≥25 kg/m2	1.620 (0.954-2.752)	1.887 (1.173-3.037)	1.205 (0.754-1.926)	1.324 (0.847-2.068)	1.141 (0.705-1.847)	1.358 (0.866-2.131)	1.057 (0.594-1.883)	1.286 (0.780-2.120)	1.645 (0.876-3.090)	1.358 (0.852-2.165)
**Total cholesterol**										
<200 mg/dL	1.000 (reference)	1.281 (0.892-1.840)	1.000 (reference)	0.967 (0.682-1.372)	1.000 (reference)	0.898 (0.634-1.273)	1.000 (reference)	0.812 (0.563-1.171)	1.000 (reference)	0.876 (0.594-1.292)
≥200 mg/dL	0.783 (0.430-1.426)	0.956 (0.581-1.572)	0.889 (0.524-1.508)	0.634 (0.392-1.027)	0.824 (0.483-1.405)	0.624 (0.384-1.014)	0.432 (0.223-0.836)	0.640 (0.385-1.064)	0.595 (0.301-1.174)	0.564 (0.340-0.935)
**Triglycerides**										
<150 mg/dL	1.000 (reference)	1.243 (0.861-1.793)	1.000 (reference)	0.873 (0.615-1.239)	1.000 (reference)	0.861 (0.606-1.224)	1.000 (reference)	1.069 (0.737-1.550)	1.000 (reference)	0.988 (0.677-1.444)
≥150 mg/dL	0.995 (0.610-1.622)	1.480 (0.966-2.269)	1.136 (0.737-1.751)	0.979 (0.644-1.490)	1.171 (0.745-1.840)	0.943 (0.621-1.432)	1.559 (0.911-2.670)	1.129 (0.736-1.733)	1.339 (0.762-2.353)	1.103 (0.727-1.675)
**HDL-C**										
≥40 (M)/≥50 (F) mg/dL	1.000 (reference)	0.992 (0.586-1.680)	1.000 (reference)	1.246 (0.751-2.067)	1.000 (reference)	1.112 (0.669-1.848)	1.000 (reference)	0.942 (0.534-1.661)	1.000 (reference)	0.668 (0.383-1.163)
<40 (M)/<50 (F) mg/dL	1.067 (0.587-1.937)	2.008 (1.127-3.576)	2.054 (1.208-3.493)	1.284 (0.767-2.150)	1.926 (1.103-3.365)	1.265 (0.747-2.144)	1.753 (0.900-3.413)	1.352 (0.755-2.419)	1.027 (0.516-2.045)	1.107 (0.620-1.977)
**LDL-C**										
<130 mg/dL	1.000 (reference)	1.418 (0.897-2.241)	1.000 (reference)	0.963 (0.618-1.501)	1.000 (reference)	0.865 (0.555-1.349)	1.000 (reference)	0.720 (0.447-1.161)	1.000 (reference)	0.779 (0.466-1.301)
≥130 mg/dL	0.820 (0.302-2.227)	0.561 (0.267-1.180)	0.785 (0.354-1.741)	0.868 (0.389-1.937)	0.639 (0.286-1.431)	0.790 (0.350-1.785)	0.756 (0.296-1.927)	0.585 (0.276-1.239)	0.428 (0.147-1.244)	0.468 (0.210-1.042)
**Homocysteine**										
<13.2 mmol/L	1.000 (reference)	1.313 (0.954-1.806)	1.000 (reference)	0.915 (0.676-1.239)	1.000 (reference)	0.792 (0.584-1.075)	1.000 (reference)	0.89 (0.643-1.231)	1.000 (reference)	0.905 (0.649-1.262)
≥13.2 mmol/L	1.658 (0.844-3.254)	2.141 (1.216-3.768)	1.625 (0.904-2.923)	0.918 (0.495-1.703)	1.346 (0.731-2.476)	1.042 (0.575-1.889)	1.053 (0.539-2.059)	1.398 (0.780-2.503)	1.195 (0.552-2.586)	1.382 (0.799-2.391)
**Vitamin B12**										
>435 pg/mL	1.000 (reference)	1.523 (0.824-2.814)	1.000 (reference)	0.594 (0.332-1.065)	1.000 (reference)	0.537 (0.303-0.952)	1.000 (reference)	0.681 (0.382-1.215)	1.000 (reference)	0.712 (0.386-1.310)
≤435 pg/mL	1.664 (0.482-5.747)	3.813 (1.644-8.845)	2.111 (0.857-5.198)	1.259 (0.500-3.173)	1.602 (0.626-4.100)	1.487 (0.605-3.658)	1.409 (0.472-4.210)	1.564 (0.700-3.497)	1.941 (0.646-5.834)	1.650 (0.677-4.023)
**Folate**										
>3.85 nmol/L	1.000 (reference)	1.291 (0.934-1.786)	1.000 (reference)	0.813 (0.595-1.110)	1.000 (reference)	0.712 (0.521-0.973)	1.000 (reference)	0.875 (0.631-1.214)	1.000 (reference)	0.859 (0.612-1.207)
≤3.85 nmol/L	2.888 (1.356-6.153)	3.836 (2.124-6.927)	3.025 (1.579-5.794)	1.964 (1.043-3.697)	2.387 (1.204-4.733)	2.070 (1.125-3.808)	2.315 (1.041-5.148)	2.553 (1.423-4.579)	2.455 (1.073-5.616)	2.572 (1.427-4.637)
**Uric acid**										

<6.5 ng/mL	1.000 (reference)	1.062 (0.775-1.453)	1.000 (reference)	0.922 (0.681-1.248)	1.000 (reference)	0.839 (0.619-1.138)	1.000 (reference)	0.942 (0.681-1.303)	1.000 (reference)	0.866 (0.620-1.210)
≥6.5 ng/mL	1.009 (0.497-2.047)	3.328 (1.870-5.923)	2.039 (1.115-3.728)	1.694 (0.919-3.123)	1.666 (0.895-3.103)	1.860 (1.011-3.420)	1.933 (0.961-3.887)	1.843 (1.022-3.325)	2.059 (0.956-4.434)	1.687 (0.957-2.972)
**Platelet**										
<296	1.000 (reference)	1.320 (0.966-1.805)	1.000 (reference)	0.852 (0.629-1.153)	1.000 (reference)	0.835 (0.616-1.131)	1.000 (reference)	1.040 (0.755-1.434)	1.000 (reference)	1.043 (0.749-1.451)
≥296	0.996 (0.475-2.091)	0.943 (0.571-1.556)	0.766 (0.405-1.450)	0.741 (0.440-1.248)	0.870 (0.446-1.696)	0.717 (0.432-1.189)	1.373 (0.681-2.768)	0.649 (0.382-1.103)	1.481 (0.683-3.212)	0.718 (0.428-1.202)
**PT**										
≤12.5 sec	1.000 (reference)	1.194 (0.848-1.682)	1.000 (reference)	0.799 (0.574-1.112)	1.000 (reference)	0.857 (0.616-1.194)	1.000 (reference)	0.818 (0.575-1.166)	1.000 (reference)	0.939 (0.655-1.347)
>12.5 sec	1.431 (0.727-2.817)	2.400 (1.321-4.359)	1.424 (0.806-2.516)	1.541 (0.768-3.095)	1.544 (0.853-2.795)	1.475 (0.770-2.825)	1.139 (0.557-2.327)	1.631 (0.889-2.991)	2.507 (1.022-6.150)	1.321 (0.757-2.306)
**Antithrombin**										
≥80 mg/dL	1.000 (reference)	1.300 (0.591-2.862)	1.000 (reference)	0.494 (0.229-1.067)	1.000 (reference)	0.566 (0.267-1.202)	1.000 (reference)	0.574 (0.254-1.295)	1.000 (reference)	1.173 (0.489-2.814)
<80 mg/dL	2.255 (0.712-7.144)	2.142 (0.791-5.800)	1.255 (0.473-3.330)	1.250 (0.471-3.316)	1.438 (0.523-3.957)	1.087 (0.419-2.825)	0.827 (0.221-3.090)	1.622 (0.584-4.506)	1.710 (0.509-5.748)	2.585 (0.816-8.187)
**HbA1C**										
<5.7 %	1.000 (reference)	1.016 (0.515-2.008)	1.000 (reference)	1.735 (0.901-3.340)	1.000 (reference)	1.671 (0.864-3.233)	1.000 (reference)	1.219 (0.626-2.373)	1.000 (reference)	0.456 (0.218-0.956)
≥5.7 %	0.801 (0.377-1.699)	1.160 (0.577-2.334)	1.367 (0.729-2.564)	1.524 (0.773-3.005)	1.474 (0.767-2.832)	1.347 (0.684-2.654)	1.856 (0.754-4.571)	1.046 (0.542-2.015)	0.486 (0.198-1.188)	0.635 (0.303-1.333)
**MCV**										
≥79 fL	1.000 (reference)	1.259 (0.942-1.684)	1.000 (reference)	0.828 (0.626-1.095)	1.000 (reference)	0.799 (0.603-1.058)	1.000 (reference)	0.912 (0.678-1.227)	1.000 (reference)	0.939 (0.691-1.275)
<79 fL	1.666 (0.128-1.631)	1.509 (0.406-5.612)	N/A	1.803 (0.493-6.602)	N/A	1.806 (0.498-6.547)	1.062 (0.203-5.551)	2.013 (0.346-1.706)	3.901 (0.414-6.734)	0.901 (0.203-4.004)
**RDW**										
≤14.5 %	1.000 (reference)	1.295 (0.964-1.741)	1.000 (reference)	0.815 (0.613-1.084)	1.000 (reference)	0.787 (0.591-1.047)	1.000 (reference)	0.888 (0.657-1.201)	1.000 (reference)	0.968 (0.709-1.323)
>14.5 %	1.721 (0.540-5.486)	1.211 (0.537-2.731)	0.629 (0.158-2.506)	1.211 (0.557-2.634)	0.629 (0.159-2.484)	1.207 (0.557-2.617)	0.779 (0.268-2.260)	1.366 (0.575-3.244)	2.404 (0.708-8.163)	0.826 (0.362-1.886)
**PDW**										
<16.5 fL	1.000 (reference)	1.270 (0.838-1.923)	1.000 (reference)	0.736 (0.494-1.098)	1.000 (reference)	0.800 (0.536-1.194)	1.000 (reference)	0.777 (0.512-1.178)	1.000 (reference)	1.177 (0.756-1.833)
≥16.5 fL	1.184 (0.501-2.799)	1.771 (0.878-3.573)	1.483 (0.746-2.951)	1.011 (0.456-2.239)	1.555 (0.751-3.220)	1.076 (0.509-2.271)	1.488 (0.573-3.862)	1.031 (0.533-1.994)	1.265 (0.532-3.008)	1.646 (0.806-3.361)
**Neutrophil**										
≤75 %	1.000 (reference)	1.284 (0.945-1.746)	1.000 (reference)	0.825 (0.615-1.108)	1.000 (reference)	0.867 (0.645-1.164)	1.000 (reference)	0.857 (0.628-1.170)	1.000 (reference)	0.961 (0.697-1.326)
>75 %	3.010 (1.261-7.183)	2.221 (1.080-4.564)	1.804 (0.829-3.928)	1.787 (0.814-3.925)	3.009 (1.231-7.356)	1.312 (0.645-2.671)	1.776 (0.700-4.505)	1.859 (0.909-3.802)	3.545 (1.247-0.073)	1.691 (0.849-3.368)

Note: PAI-1, plasminogen activator inhibitor-1; AOR, adjusted odds ratio; CI, confidence interval; FBS, fasting blood sugar; BMI, body mass index; HDL-C, high-density lipoprotein cholesterol; M, male; F, female; LDL-C, low-density lipoprotein cholesterol; PT, prothrombin time; HbA1C, hemoglobin A1C; MCV, mean corpuscular volume; RDW, red blood cell distribution width; PDW, platelet distribution width; N/A, not applicable.
AOR was adjusted by age, sex, hypertension, diabetes mellitus, hyperlipidemia, and smoking status.

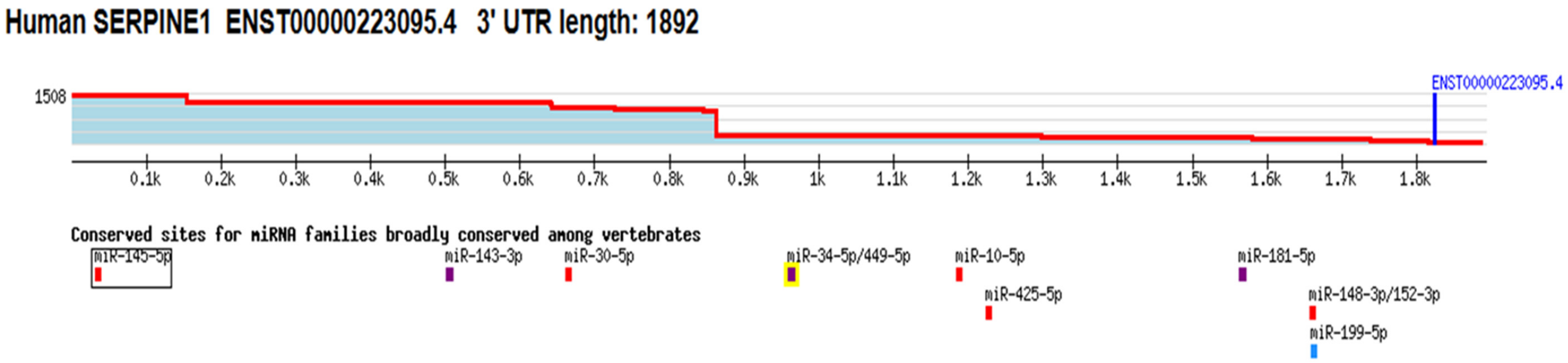

Figure S1. miRNAs that bind to SERPINE1 predicted using TargetScan.

Gene name	miRNA name	Experiments throughput	Publications	Cell lines	Tissues	Pred. Score
SERPINE1	hsa-miR-30c-5p	low: 5 high: 1	3	3	3	-
SERPINE1	hsa-miR-99a-5p	low: 3 high: 0	1	1	1	-
SERPINE1	hsa-miR-204-5p	low: 2 high: 1	1	2	1	-
SERPINE1	hsa-miR-301a-3p	low: 2 high: 0	1	1	1	-
SERPINE1	hsa-miR-145-5p	low: 1 high: 2	2	2	2	-
SERPINE1	hsa-miR-148a-3p	low: 1 high: 2	2	3	2	0.832
SERPINE1	hsa-miR-138-5p	low: 1 high: 1	2	2	2	-
SERPINE1	hsa-miR-143-3p	low: 0 high: 4	2	4	2	-
SERPINE1	hsa-miR-342-3p	low: 0 high: 4	2	4	2	0.675
SERPINE1	hsa-miR-335-5p	low: 0 high: 4	2	3	2	-
SERPINE1	hsa-miR-224-5p	low: 0 high: 3	2	3	2	0.764
SERPINE1	hsa-miR-10a-5p	low: 0 high: 3	2	3	2	0.796
SERPINE1	hsa-miR-17-5p	low: 0 high: 3	1	3	2	-
SERPINE1	hsa-miR-181a-5p	low: 0 high: 3	1	3	2	-
SERPINE1	hsa-miR-196b-5p	low: 0 high: 3	2	3	2	0.743

Figure S2. miRNAs that bind to SERPINE1 predicted using Tarbase v8.

Table S3. List of primers used for real-time PCR.

miRNAs	SNP	Sequence	Location
miR-30c	rs928508 A>G	Forward 5'-AGT GGT GGG GCT AAA AC-3'<br>Reverse 5'-GCT TGT CAG TAA ATG CTG TC-3'	chromosome 1/ miR-30c: 500bp downstream
miR-143	rs41291957 G>A	Forward 5'-AGG TCA AGG TTT GGT CCT G-3'<br>Reverse 5'-TCC CAA CTG ACC AGA GAT G-3'	chromosome 5 / miR-143: 2kb upstream
	rs4705342 T>C	Forward 5'-AGG AGT GGC AGA AGA AAG-3'<br>Reverse 5'-TGG AAT TAT TTA ATA TGT TGA CCT AC-3'	chromosome 5 / miR-143: 2kb upstream
miR-145	rs353291 T>C	Forward 5'-AAC TAG TAA ACA CAC ATG AAT TC-3'<br>Reverse 5'-GGT AGA GAT GCC ACA AGA GA -3'	chromosome 5 / miR-145: 500bp downstream
miR-181a2	rs10760371 T>G	Forward 5'- GCT AAG ATC TGA GCA GAG C-3'<br>Reverse 5'- CCT CTT AGC ATG CTA AAA CC-3'	chromosome 9/miR-181a2: 2kb upstream
